# Supplementary material for: The neurocomputational link between defensive cardiac states and approach-avoidance arbitration under threat
Source: Commun Biol. 2024 May 16;7:576. doi: 10.1038/s42003-024-06267-6 (PMC11099143; doi:10.1038/s42003-024-06267-6)
Supplement: Supplementary file 2 — Supplementary Information [file 42003_2024_6267_MOESM2_ESM.pdf]

SUPPLEMENTARY INFORMATION

Supplementary Table 1. Coordinates and statistics of positive and negative correlations between BOLD and reward/threat magnitudes during the anticipation screen

Region	Side	x, mm	y, mm	z, mm	Cluster size, mm^3	Peak T	*p* value
Reward – positive							
Occipital cortex	L/R	-16	-102	-2	58,808	-	< .001
Amygdala	L	-20	-2	-14	1,672	-	= .003
Precentral gyrus	L	-36	-22	60	1,576	-	< .001
Supplemental motor area	L/R	2	-8	58	4,048	-	< .001
Ventral striatum	L	-6	10	-4	-	4.57	= .003*
	L	-16	10	-12	-	3.60	= .050*
	R	8	8	-2	-	5.26	< .001*
	R	10	12	-10	-	4.47	= .004*
vmPFC	R	4	64	-2	-	4.16	= .059*
Reward – negative							
Lateral orbitofrontal cortex	R	38	42	-10	1,120	-	= .024
Threat – positive							
Occipital cortex	L/R	-18	-78	-12	30,712	-	< .001
SMA/dACC	L/R	10	10	46	11,872	-	< .001
	L	-2	2	44	-	4.68	= .025*
	R	10	10	44	-	5.99	< .001*
	R	8	26	30	-	4.95	= .011*
	R	14	20	32	-	4.79	= .018*
	R	10	24	34	-	4.76	= .020*
Insular cortex	L	-42	16	2	3,512	-	< .001
	R	34	18	-8	7,000	-	< .001
Postcentral gyrus	L	-44	-26	52	4,744	-	< .001
dlPFC	R	34	32	36	5,568	-	< .001
	L	-32	46	24	2,240	-	< .001
Precuneus	L/R	6	-54	54	6,528	-	< .001
Temporoparietal junction	L	-58	-48	42	3,032	-	< .001
	R	62	-44	32	4,504	-	< .001
Intraparietal sulcus	L	-18	-74	48	1,240	-	< .001
Threat – negative							
vmPFC	L/R	-4	50	-10	2,016	-	< .001
Rew-by-Thr – negative							
Occipital cortex	L/R	-6	-80	-6	3,464	-	< .001

All coordinates are defined in MNI152 space. All listed statistics are significant at p < .05 FWE-corrected at the cluster-level (whole-brain) or peak-level small volume corrected (FWE-SVC, predefined ROIs, indicated with asterisks ''). dACC: dorsal anterior cingulate cortex; SMA: supplemental motor area; dlPFC: dorsolateral prefrontal cortex; vmPFC: ventromedial prefrontal cortex.*

Supplementary Table 2. Coordinates and statistics of (de)activations as a function of passive and active approach vs. avoidance choices during the anticipation screen

Region	Side	x, mm	y, mm	z, mm	Cluster size, mm^3	Peak T	*p* value
Approach > Avoid							
Occipital cortex	L	-20	-98	2	6,216	-	< .001
	R	28	-98	-6	2,208	-	< .001
Hippocampus/PHG/AMY	R	22	-10	-14	16,192	-	< .001
Amygdala	L	-18	-2	-22	-	4.37	= .005*
	L	-28	-6	-12	-	4.09	= .011*
	L	-16	-4	-18	-	3.98	= .015*
	L	-16	-2	-14	-	3.95	= .016*
	L	-24	0	-12	-	3.79	= .024*
	L	-12	-2	-16	-	3.53	= .048*
	R	24	-8	-14	-	5.44	< .001*
	R	30	-4	-18	-	4.82	= .001*
	R	20	-2	-18	-	4.14	= .009*
	R	26	4	-28	-	3.78	= .025*
	R	32	4	-26	-	3.27	= .090*
Precentral gyrus	L	-32	24	62	6,384	-	< .001
	L	-54	2	40	992	-	= .036
	R	56	6	40	960	-	= .041
Supplemental motor area	L/R	2	-16	64	8,544	-	< .001
Parietal operculum	R	56	2	6	944	-	= .044
vmPFC	L/R	-4	64	-6	5,504	-	< .001
Ventral striatum	L	-6	8	-4	-	5.72	< .001*
	L	-8	8	-10	-	4.85	= .001*
	R	8	8	-4	-	5.54	< .001*
	R	6	12	-4	-	5.54	< .001*
BNST	L	-6	4	-4	-	3.62	= .010*
	L	-4	0	0	-	3.08	= .040*
	R	8	4	-4	-	4.05	= .003*
	R	6	0	-2	-	3.71	= .008*
Approach < Avoid							
Precuneus	R	6	-56	54	3,520	-	< .001
Active > Passive							
Postcentral gyrus	L	-34	-22	44	4,728	-	< .001
Thalamus	L/R	4	-18	0	872	-	= .030
$Choice_{Passive}$ > $Choice_{Active}$							
Lingual gyrus	L/R	-2	-66	6	960	-	= .021
Supplemental motor area	L/R	-4	-4	64	4,416	-	< .001
Postcentral gyrus	L	-40	-22	56	1,432	-	= .002
$Choice_{Passive}$ < $Choice_{Active}$							
Occipital cortex	L	-16	-92	-8	2,016	-	< .001
	R	20	-90	-4	2,464	-	< .001

All coordinates are defined in MNI152 space. All listed statistics are significant at p < .05 FWE-corrected at the cluster-level (whole-brain) or peak-level small volume corrected (FWE-SVC; predefined ROIs, indicated with asterisks ''). PHG: parahippocampal gyrus; AMY: amygdala; vmPFC: ventromedial prefrontal cortex; ACC: anterior cingulate cortex; BNST: bed nucleus of the stria terminalis.*

Similar neural effects for anticipation of passive vs. active approach-avoidance choices

We explored whether the observed neural approach-avoidance circuit (see main text) was differentially involved in passive vs. active approach-avoidance choices. The choice-by-response interaction indicated a significant effect in the SMA (*p* < .001 cluster-level FWE), left postcentral gyrus (*p* = .002 cluster-level FWE), left BNST (*p* = .005 peak-voxel FWE-SVC), and right ventral striatum (*p* = .005 peak-voxel FWE-SVC). In these areas, the approach-vs-avoid effect on BOLD was larger for passive compared to active responses, potentially signaling modulation of decision-related neural activity by motor preparation (**Supplementary Table 2**).

Neural effects reflect decision (and not response) related changes in brain activity

To verify whether our neural effects (main-text **Figure 3**) reflect decision-related rather than response-related changes in brain activation, we performed a finite impulse response (FIR) analysis to inspect the time course of the BOLD signal without assumptions on the shape of the hemodynamic response function (HRF). We focused on three ROIs that showed an effect for the voxel-wise approach vs. avoid contrast described above: the bilateral amygdala (AMY), ventral striatum (vStr), and vmPFC (**Supplementary Figure 1**). Three-way repeated measures ANOVAs revealed significant main effects of time and choice on BOLD in the amygdala and ventral striatum (time_{AMY}: F(1.1045, 62.9558) = 4.259, *p* = .0137, η_p^2 = .069; $\text{time}_{\text{vStr}}$: F(0.9509, 54.2004) = 22.028, *p* < .001, η_p^2 = .278; $\text{choice}_{\text{AMY}}$: F(0.36816, 20.9853) = 12.205, p < .001, η_p^2 = .176; $\text{choice}_{\text{vStr}}$: F(0.3168, 18.0668) = 8.6332, *p* = .0046, η_p^2 = .132), a marginally significant main effect of choice in the vmPFC (F(0.31522, 17.96754) = 3.8532, *p* = .054, η_p^2 = .063), and an interaction effect between time and choice for all three ROIs (AMY: F(1.1045, 62.9558) = 5.0339, *p* = .003, η_p^2 = .081; vStr: F(0.9509, 54.2005) = 9.4897, *p* < .001, η_p^2 = .142; vmPFC: F(0.94566, 53.90262) = 7.8983, *p* < .001, η_p^2 = .121). More specifically, in all three regions the choice effect on BOLD was most pronounced in the bin spanning 4.5 – 6 seconds after stimulus onset (AMY: t(57) = 4.1693, *p* < .001 ; vStr: t(57) = 4.6589; *p* < .001; vmPFC: t(57) = 3.5415; *p* < .001). In none of the regions there was a significant main effect of, nor an interaction effect involving, active vs. passive responses. These results further support the suggestion that the voxel-wise differences described above reflect value-based decision (and not response) related changes in BOLD response patterns (**Supplementary Figure 1**).

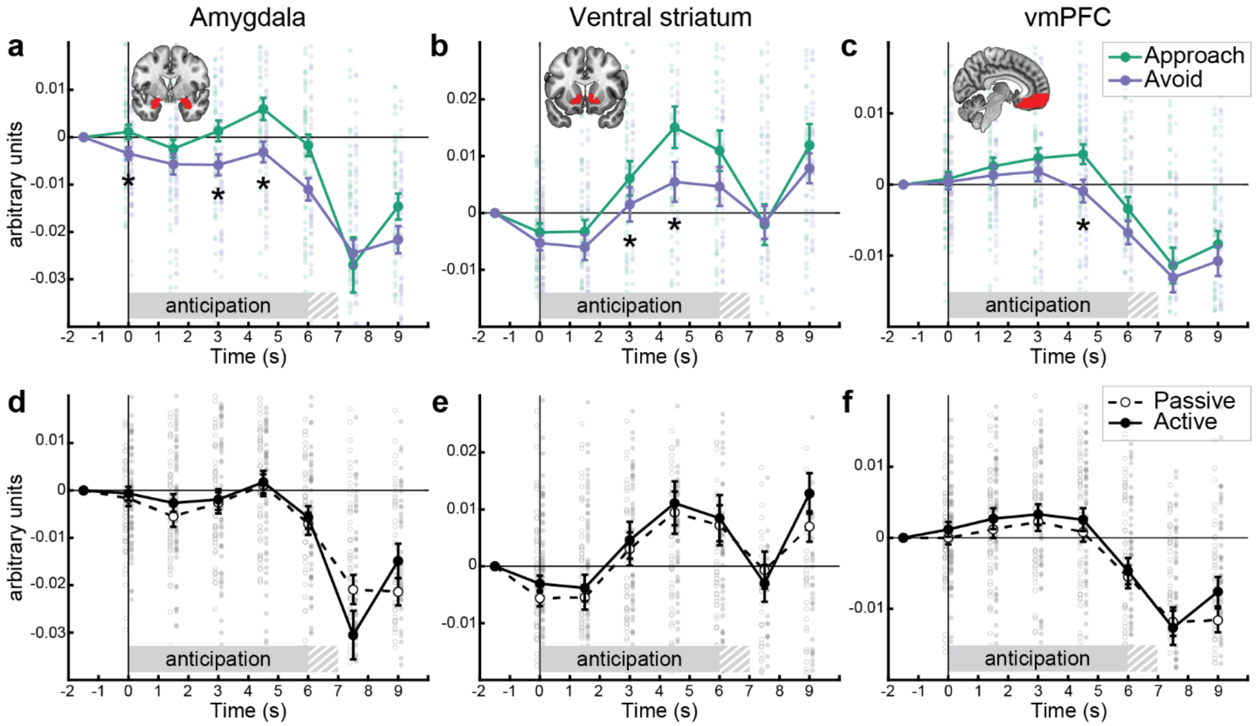

Supplementary Figure 1. Time course trajectories of BOLD activity in the amygdala, ventral striatum, and vmPFC during the anticipation screen. *Stronger BOLD activity in approach compared to avoid trials in the amygdala (**a**), ventral striatum (**b**), and vmPFC (**c**) occur in time bins during the anticipation screen. Additionally, BOLD activity did not significantly differ as a function of subsequent active vs. passive responses (**d-f**). Each bin spans a time window of 1.5 s (e.g., bin 1 ranges from t = -1.5 to t = 0). Responses are plotted relative to the onset of the stimulus screen and baseline corrected relative to the first bin, indicating that neural effects are strongest during decision rather than the response. Small, semi-transparent dots represent individual participant data (n=58 per condition). Error bars indicate +/- 1 SEM. Gray-white striped shaded areas reflect partial overlap between stimulus and target movement screens across different trials (i.e., movement window onset was uniformly jittered between 6 – 7 s). Asterisks indicate significant (p < .05) follow-up t-tests. vmPFC: ventromedial prefrontal cortex. Anatomical ROIs used to extract the BOLD signal are plotted in red.*

Formal comparison of freezing and base models

We compared the fit of the three freezing models amongst themselves and with the base model. We used formal model comparison to find out to which of these models seems fits the choice behavior best. The comparison showed that while all models performed very similarly (i.e., the standard errors of the model comparison metrics between models overlapped), the *aversive value* model outperformed the base model as well as the other freezing models (i.e., the ‘looic’ was lowest, see **Supplementary Table 3**).

Supplementary Table 3. Model comparison results of base and freezing models

**Model**	looic	se_{looic}	$ELPD_{diff}$	se_{diff}
Base	6049.105	110.4498	-.04720	2.4694
Route 1 (AV)	6048.161	110.6306	0	0
Route 2 (VC)	6049.457	110.5524	-0.6479	1.9312
Route 3 (AI)	6048.531	110.5737	-0.1852	3.2587

Lower model fit estimates (looic) indicate better fit. ELPD is the theoretical expected log pointwise predictive density for a new data set, estimated through leave-one-out (loo) cross validation. These estimates are compared between models using the $ELPD_{diff}$ metric, which reflects each model's ELPD relative to the best fitting model (in this case AV; more negative $ELPD_{diff}$ values indicate worse fit). The model with the best model fit is highlighted in bold.s

Supplementary Table 4. Coordinates and statistics of positive and negative correlations between BOLD and model-based approach-avoidance during the anticipation screen

Region	Side	x, mm	y, mm	z, mm	Cluster size, mm^3	Peak T	*p* value
Base model – positive							
Occipital cortex	L	-18	-98	0	8,328	-	< .001
	R	4	-82	-10	2,720	-	< .001
vmPFC	L/R	4	64	-2	4,416	-	< .001
Ventral striatum	L	-8	10	-8	-	4.96	= .001*
	R	6	10	-4	-	4.23	= .009*
	R	10	10	-8	-	3.97	= .019*
Amygdala	R	32	-2	-18	-	3.87	= .019*
Base model – negative							
Precuneus	R	8	-52	52	2,136	-	<.001
Superior frontal gyrus	R	20	4	62	1,448	-	= .008
AV – negative							
Middle temporal cortex	R	56	4	-30	1,208	-	= .007
Paracentral lobule	R	6	-30	72	2,432	-	< .001
Inferior frontal cortex	L	-56	14	26	1,344	-	= .004
dlPFC	L	-34	4	62	1,128	-	= .010
Amygdala	L	-16	-2	-12	-	3.33	= .086*
	R	34	2	-22	-	3.87	= .022*
	R	26	0	-14	-	3.36	= .081*
	R	30	-2	-26	-	3.28	= .096*
AV – negative-avoidance							
Amygdala	L	-2230	2	-16	-	3.58	= .045*
	R	30	-2	-26	-	4.02	= .014*
	R	26	2	-22	-	3.92	= .018*
	R	26	0	-12	-	3.77	= .027*
VC – positive							
dmPFC (dACC/SMA)	L/R	2	-6	70	17,664	-	< .001
Postcentral gyrus	L	-48	-12	28	3,064	-	< .001
Middle occipital cortex	L	-24	-72	24	1,640	-	= .002
Inferior temporal cortex	R	52	-10	26	2,016	-	< .001
Caudate nucleus	R	-16	-2	22	1,008	-	= .025
Cerebellum	L	-42	-56	-24	2,256	-	< .001
Fusiform gyrus	R	40	-60	-18	688	-	= .035
VC – positive-approach							
dmPFC (dACC/SMA)	L/R	-8	14	34	1,472	-	= .004
dACC	L/R	-8	14	34	-	4.93	< .001*

All coordinates are defined in MNI152 space. All listed statistics are significant at p < .05 FWE-corrected at the cluster-level (whole-brain) or peak-level small volume corrected (SVC; predefined ROIs, indicated

with asterisks ‘’). For ROIs only, peak voxels with* $.05 < p_{FWE} < .1$ *are also listed. dmPFC: dorsomedial prefrontal cortex; dACC: dorsal anterior cingulate cortex; SMA: supplemental motor area; vmPFC: ventromedial prefrontal cortex; dlPFC: dorsolateral prefrontal cortex.*
