# Supplementary material for: The neurocomputational link between defensive cardiac states and approach-avoidance arbitration under threat
Source: Commun Biol. 2024 May 16;7:576. doi: 10.1038/s42003-024-06267-6 (PMC11099143; doi:10.1038/s42003-024-06267-6)
Supplement: Supplementary file 3 — Reporting summary [file 42003_2024_6267_MOESM3_ESM.pdf]

nature portfolio

Corresponding author(s): Felix Klaassen

Last updated by author(s): Apr 12, 2024

Reporting Summary

Nature Portfolio wishes to improve the reproducibility of the work that we publish. This form provides structure for consistency and transparency in reporting. For further information on Nature Portfolio policies, see our Editorial Policies and the Editorial Policy Checklist.

Statistics

For all statistical analyses, confirm that the following items are present in the figure legend, table legend, main text, or Methods section.

n/a	Confirmed	
☐	☒	The exact sample size (n) for each experimental group/condition, given as a discrete number and unit of measurement
☐	☒	A statement on whether measurements were taken from distinct samples or whether the same sample was measured repeatedly
☐	☒	The statistical test(s) used AND whether they are one- or two-sided<br>*Only common tests should be described solely by name; describe more complex techniques in the Methods section.*
☒	☐	A description of all covariates tested
☐	☒	A description of any assumptions or corrections, such as tests of normality and adjustment for multiple comparisons
☐	☒	A full description of the statistical parameters including central tendency (e.g. means) or other basic estimates (e.g. regression coefficient) AND variation (e.g. standard deviation) or associated estimates of uncertainty (e.g. confidence intervals)
☐	☒	For null hypothesis testing, the test statistic (e.g. F, t, r) with confidence intervals, effect sizes, degrees of freedom and P value noted<br>*Give P values as exact values whenever suitable.*
☐	☒	For Bayesian analysis, information on the choice of priors and Markov chain Monte Carlo settings
☐	☒	For hierarchical and complex designs, identification of the appropriate level for tests and full reporting of outcomes
☐	☒	Estimates of effect sizes (e.g. Cohen's d, Pearson's r), indicating how they were calculated

Our web collection on statistics for biologists contains articles on many of the points above.

Software and code

Policy information about availability of computer code

Data collection	Behavioral task data was acquired using MATLAB 2016 and the PsychToolbox extension. MRI data were acquired using a SIEMENS MAGNETOM PrismaFit scanner. Physiological data were acquired using hardware and software from Brain Vision and Brain Products.
Data analysis	All physiological and MRI data analyses were performed in MATLAB (including the SPM12 toolbox). Behavioral models were run in R (4.0.2) using RStudio (2022.12.0).

For manuscripts utilizing custom algorithms or software that are central to the research but not yet described in published literature, software must be made available to editors and reviewers. We strongly encourage code deposition in a community repository (e.g. GitHub). See the Nature Portfolio guidelines for submitting code & software for further information.

Data

Policy information about availability of data

All manuscripts must include a data availability statement. This statement should provide the following information, where applicable:

- Accession codes, unique identifiers, or web links for publicly available datasets
- A description of any restrictions on data availability
- For clinical datasets or third party data, please ensure that the statement adheres to our policy

The source data supporting the findings of this study are available in the Radboud Data Repository (data.ru.nl) under the identifier di.dccn.DSC_3023009.03_522, at https://doi.org/10.34973/tvyt-h588.

Research involving human participants, their data, or biological material

Policy information about studies with human participants or human data. See also policy information about sex, gender (identity/presentation), and sexual orientation and race, ethnicity and racism.

Reporting on sex and gender	Self-reported sex (male/female/intersex) was recorded as part of the data collection, but not used for any data analyses as it was not a variable of interest for the study. Sex was merely recorded to characterize the study sample.
Reporting on race, ethnicity, or other socially relevant groupings	We did not record any information regarding race/ethnicity in this study.
Population characteristics	See above
Recruitment	Participants were recruited through an online participant-recruitment platform on which they could sign up for studies. As a consequence, the study sample mostly consists university bachelor/master students studying and/or living in Nijmegen (The Netherlands).
Ethics oversight	All research activities were carried out in accordance with the Declaration of Helsinki, approved by the local ethics committee (Ethical Reviewing Board CMO/METC [Institutional Research Review Board] Arnhem-Nijmegen, CMO 2014/288), and all ethical regulations relevant to human research participants were followed.

Note that full information on the approval of the study protocol must also be provided in the manuscript.

Field-specific reporting

Please select the one below that is the best fit for your research. If you are not sure, read the appropriate sections before making your selection.

☐ Life sciences ☒ Behavioural & social sciences ☐ Ecological, evolutionary & environmental sciences

For a reference copy of the document with all sections, see nature.com/documents/nr-reporting-summary-flat.pdf

Behavioural & social sciences study design

All studies must disclose on these points even when the disclosure is negative.

Study description	This study follows a quantitative, experimental design.
Research sample	The research study mostly consists of bachelor/master students attending the Radboud University in Nijmegen (The Netherlands). The sample was aged 18 - 34, [M±SD = 24.17±3.43], 41 females.
Sampling strategy	We used a convenience sample. The a-priori sample size rationale was pre-registered as follows:<br>Our set sample size was not based on a calculation but chosen based on both empirical and resource-related constraints (Lakens, 2021). We informed our sample by two related fMRI studies that had N=18 (Hermans, Henckens, Roelofs, & Fernández, 2013), N=24 (Park et al., 2011), and our own previous behavioral study with the same paradigm where N=42 (Klaassen et al., 2021). To account for potential inflated effect-sizes (Button et al., 2013) and to increase the ability to reliably replicate the previous behavioral findings (e.g., Camerer et al., 2018) we opted for a larger sample size. Taking the available resources (time and money) into account we compromised for a sample size of 60 participants.
Data collection	Experimental task data were collected using pc's running on Windows 7 running MATLAB; fMRI data was collected using software and hardware provided by SIEMENS; physiological data were acquired using hardware and software from Brain Products and Brain Vision; an online data collection program (CastorEDC) was used to collect demographic data. The main researcher and at most one other researcher were present during the experiment. The researcher was fully aware of the study hypotheses and experimental conditions.
Timing	Data collection started in October 2020 and ended in January 2022.
Data exclusions	Sixty-seven healthy volunteers completed the study. After data collection, 9 participants were excluded from data analysis due to an imaging artifact (n=3), too little variance in choice behavior (n=3), unusable heart rate data (n=2), or falling asleep during the experiment (n=1), leading to a sample size of 58 participants.<br><br>For these subjects' data, as pre-registered, we only included trials with long anticipation-to-movement screen intervals in our analyses (i.e., 150 trials per participant). For 8 (out of 58) subjects we only had usable data of 2 out of 3 runs (i.e., 100 trials), due to a lack of observations in our cells of interest for fMRI analysis (i.e., passive and active approach-avoidance choices). For consistency, we used the same data set across all analyses. Additionally, we excluded trials with poor heart rate data, or response times below 200 ms (i.e., excluding a further 304 trials (±3.7%) from the data set). For all analyses involving response times, only trials with active responses (i.e., button-presses) were used (±48.7% of the data set).
Non-participation	No participants dropped out of the study.

Randomization | Participants were not randomly allocated to experimental groups (within-subjects design).

Reporting for specific materials, systems and methods

We require information from authors about some types of materials, experimental systems and methods used in many studies. Here, indicate whether each material, system or method listed is relevant to your study. If you are not sure if a list item applies to your research, read the appropriate section before selecting a response.

Materials & experimental systems

n/a	Involved in the study
☒	☐ Antibodies
☒	☐ Eukaryotic cell lines
☒	☐ Palaeontology and archaeology
☒	☐ Animals and other organisms
☒	☐ Clinical data
☒	☐ Dual use research of concern
☒	☐ Plants

Methods

n/a	Involved in the study
☒	☐ ChIP-seq
☒	☐ Flow cytometry
☐	☒ MRI-based neuroimaging

Magnetic resonance imaging

Experimental design

Design type: Event-related task design

Design specifications: Each participant completed 3 runs of 62 trials 186 (trials in total). Trial durations varied between 2.7 to 9.2 seconds, inter-trial interval durations varied between 9 - 11 seconds.

Behavioral performance measures: Button pressed (yes/no), response time (if button pressed). These were analyzed on a trial-by-trial level (plots show mean +- 1 standard error).

Acquisition

Imaging type(s): Functional and structural

Field strength: 3T

Sequence & imaging parameters: T2*-weighted BOLD-fMRI was acquired using a multiband sequence (68 axial slices, TR = 1500 ms, TE = 28 ms, flip angle = 75, multiband acceleration factor = 4, interleaved slice acquisition, slice thickness = 2 mm, voxel size = 2 mm isotropic, phase encoding direction = A>>P, bandwidth = 2290 Hz/Px, echo spacing = 0.54 ms, phase partial fourier = 7/8, FOV = 210 x 210).
A field map was acquired to correct for distortions due to structural magnetic field inhomogeneities (68 slices, TR = 435 ms, TE1 = 2.20 ms, TE2 = 4.66 ms, flip angle = 60).
One anatomical image per participant (1 mm isotropic) was acquired using a single-shot T1-weighted magnetization-prepared rapid gradient-echo sequence (MP-RAGE; acceleration factor of 2 [GRAPPA method], TR = 2300 ms, TE = 3.03 ms, flip angle = 8, 192 sagittal slices, FOV = 256 x 256 x 192 mm).

Area of acquisition: Whole-brain scans were acquired.

Diffusion MRI: ☐ Used ☒ Not used

Preprocessing

Preprocessing software: All raw MRI images were converted to nifti format and then preprocessed in SPM12 (Statistical Parametric Mapping; Wellcome Trust Centre for 694 Neuroimaging, London, UK). Functional images were realigned and unwarped using the field-map based voxel displacement map, coregistered to the anatomical image using maximization-based rigid-body registration, normalized to MNI152 space (Montreal Neurological Institute), and spatially smoothed with a Gaussian kernel of 5 mm full width at half maximum.

Normalization: Functional images were normalized using subjects' T1 anatomicals for alignment (non-linear transformation using 7th degree B-spline interpolation).

Normalization template: Subject-wise data were normalized to the MNI152 template.

Noise and artifact removal: Six rigid-body realignment parameters were added to the GLM analyses as nuisance regressors.
To correct for cardiac and respiratory noise in the BOLD signal, we applied the same procedure as described by de Voogd et al. The preprocessed pulse and respiration measures were used for retrospective image-based correction (RETROICOR) of

physiological noise artifacts in BOLD-fMRI data. Raw pulse and respiratory data were used to specify fifth-order Fourier models of the cardiac and respiratory phase-related modulation of the BOLD signal, yielding 10 nuisance regressors for cardiac noise and 10 for respiratory noise. Additional regressors were calculated for heart rate frequency, heart rate variability, (raw) abdominal circumference, respiratory frequency, respiratory amplitude, and respiration volume per unit time, yielding a total of 26 RETROICOR regressors.

Volume censoring

We excluded voxels outside of the brain (i.e., gray matter, white matter, and CSF were kept; based on the MNI152 template).

Statistical modeling & inference

Model type and settings

Unless specified otherwise, all regressors were temporally convolved with the hemodynamic response function (HRF) included in SPM12 (Statistical Parametric Mapping; Wellcome Trust Centre for Neuroimaging, London, UK). Additionally, we included six movement parameter regressors (3 translations, 3 rotations), 26 RETROICOR regressors, high-pass filtering (1/128 Hz cutoff), and AR(1) serial correlation corrections into all models.

Effect(s) tested

We created and tested contrasts for approach/avoidance choices, money/shock levels (parametric regressors), and model-based predicted approach probabilities (parametric regressors). First-level contrast maps were entered into second-level one-sample t-tests.

Specify type of analysis: ☐ Whole brain ☐ ROI-based ☒ Both

Anatomical location(s)

Anatomical locations were determined used the AAL3 (Automated Anatomical Labeling 3) atlas.

Statistic type for inference

(See Eklund et al. 2016)

For whole-brain statistics only, we will report activations that are significant at the cluster level ($p < .05$ FWE-corrected, at an initial cluster-forming threshold of $p < .001$ uncorrected). For analyses within regions of interest (ROIs) we use peak-level correction ($p < .05$ FWE-corrected).

Correction

We used FWE-correction across all analyses (see above).

Models & analysis

n/a	Involved in the study
☒	☐ Functional and/or effective connectivity
☒	☐ Graph analysis
☒	☐ Multivariate modeling or predictive analysis
